# Effect of Ecological Restoration on Body Condition of a Predator

**DOI:** 10.1371/journal.pone.0133551

**Published:** 2015-07-30

**Authors:** Daniel González-Tokman, Cristina Martínez-Garza

**Affiliations:** 1 Centro de Investigación en Biodiversidad y Conservación, Universidad Autónoma del Estado de Morelos. Cuernavaca, Morelos, México; 2 Consejo Nacional de Ciencia y Tecnología, Cátedras CONACYT, México, D. F., México; Scientific Research Centre, Slovenian Academy of Sciences and Arts, SLOVENIA

## Abstract

Ecological restoration attempts to recover the structure and function of ecosystems that have been degraded by human activities. A crucial test of ecosystem recovery would be to determine whether individuals in restored environments are as healthy as those in conserved environments. However, the impact of restoration on physiology of terrestrial animals has never been tested. Here, we evaluated the effect of two restoration methods on body condition measured as body size, body mass, lipid and muscle content of the spider *Nephila clavipes* in a tropical dry forest that has suffered chronic disturbance due to cattle grazing. We used experimental plots that had been excluded from disturbance by cattle grazing during eight years. Plots were either planted with native trees (i. e. maximal intervention), or only excluded from disturbance (i. e. minimal intervention), and were compared with control conserved (remnants of original forest) and disturbed plots (where cattle is allowed to graze). We predicted (1) better body condition in spiders of conserved and restored sites, compared to disturbed sites, and (2) better body condition in plots with maximal intervention than in plots with minimal intervention. The first prediction was not supported in males or females, and the second prediction was only supported in females: body dry mass was higher in planted than in conserved plots for spiders of both sexes and also higher that in disturbed plots for males, suggesting that plantings are providing more resources. We discuss how different life histories and environmental pressures, such as food availability, parasitism, and competition for resources can explain our contrasting findings in male and female spiders. By studying animal physiology in restoration experiments it is possible to understand the mechanistic basis of ecological and evolutionary processes that determine success of ecological restoration.

## Introduction

When a forest is degraded by human activities, ecological restoration is the most promising strategy to recover its structural and functional integrity [Society of Ecological Restoration, 2004; http://www.ser.org; [1]]. Restoration can include minimal intervention, when the disturbing agent is detected and eliminated to allow natural succession to take place, or maximal intervention, when practitioners introduce native tree species in plantings [2]. Depending on the situation, different levels of intervention to restore ecosystems can be successful and cost effective [3]. Many ecological restoration programs employ trial and error reforestation techniques to attempt to re-create lost habitats [4]. However, the only way to deduce the causes of success or failure of a restoration program, and to determine the best restoration methods is by carrying out experiments in which different restoration treatments are tested simultaneously in replicated plots [4–7].

Success of a restoration program can be evaluated in different ways depending on the program goals [5]. From an ecological perspective, restoration aims to recover composition, ecosystem function and stability, and the landscape context of a disturbed area [1]. Most experimental studies on restoration have focused on the recovery of community structure and composition, biodiversity, nutrient cycling, or abiotic measurements [8]. However, one key element of ecosystem function, the physiology of individuals, has received little attention in the context of restoration, despite its role in mediating organism responses to the environment, and its importance for shaping an ecosystem’s structure [9–11].

Physiology is largely a reflection of habitat quality and, acting at an organism level, leads to important changes at population and community levels [10,12,13]. Therefore, implementing physiological studies in restoration efforts is key to evaluate, plan, and execute restoration programs [9,14]. Compared to classical ecological techniques used to monitor restoration programs, such as population dynamics or community structure [6,15], physiological tools can detect subtle responses of organisms on a shorter time scale, and can help to elicit and understand causal relationships among environmental conditions, organism responses, evolution, and recovery processes [9,16,17].

During the process of ecosystem recovery, generalist species, which have broad nutritional and microclimatic ranges, respond physiologically to new environmental conditions and can be found in sites in different stages of recovery [18]. However, the physiological plasticity allowing generalist species to tolerate variable environmental conditions implies high energetic costs [19]. This property makes them ideal study systems to evaluate habitat quality in restored ecosystems. Predators can be especially sensitive to the new conditions, as they depend on prey availability and quality to live and reproduce [20,21]. Moreover, predators have the ability to choose their diets adaptively to compensate for specific nutritional imbalances, such as lipids or proteins [22,23], but this ability may be limited by resource availability in disturbed sites compared to restored or conserved environments.

Resource availability defines an individual’s body condition [24,25]. Individuals in good condition in terms of nutrient storage are considered ‘healthy’ because they tolerate longer periods of fasting, are more immunocompetent, and ultimately have higher mating success and fecundity than individuals in poor nutritional condition [24]. Despite the existence of high food availability in certain environments, resource availability is assumed to be scarce in nature [26]. Thus, higher nutrient storage is usually assumed to be advantageous [24] but see [27]. As body condition can be measured using different traits, those that depend on habitat quality are of particular interest in the field of conservation physiology, given that they reflect the intensity of environmental stressors [12,28]. Nutritional ecology analyses of body nutrient content, such as lipids or proteins, can help to demonstrate which macronutrients drive an individual’s condition in response to disturbance and ecosystem recovery, and are therefore recommended in conservation physiology [26,29].

In the present study we evaluated the effect of two levels of intervention in restoration: minimal (exclusion of disturbance) and maximal intervention (plantings) on the body condition of a generalist predator, the native golden orb-web spider, *Nephila clavipes*, in a tropical dry forest of Mexico. We measured spider size, body mass, lipid content and muscle mass in plots that were excluded from disturbance for eight years, and compared these measurements with control conserved and disturbed sites. We chose such condition measurements because they provide a mechanistic basis for individual survival and reproduction, and can drive population changes [30]. This nutritional ecology approach in conservation physiology has been difficult to apply to vertebrates because of the complexity of estimating energy budgets [28,30], but we overcame this limitation by using an invertebrate. We predicted better body condition in spiders from conserved sites and restoration plantings compared to sites under minimal intervention and those under disturbance. To our knowledge, this study represents the first evidence of the effect of ecological restoration on animal physiology in a terrestrial ecosystem.

## Methods

### Ethical statement

We confirm that official permission was obtained in 2005 from Secretaría del Medio Ambiente y Recursos Naturales, Mexico (SEMARNAT; Ministry of the Environment and Natural Resources permit SGPA/DGVS 07808), and has been renewed since then. The species used in this study (*Nephila clavipes*) is not an endangered or protected species under CITES regulations.

### Study species

The present study was carried out with adult male and female golden orb-web spiders, *Nephila clavipes* (Araneae: Nephilidae). This species shows marked sexual size dimorphism, with females being up to four times longer and one hundred times heavier than males [31] (see **results**). During adulthood, females build relatively permanent webs for capturing prey; males do not build webs, but instead kleptoparasitize female webs for feeding, sometimes eating prey rejected by females [32]. This spider species is considered a generalist predator given that accepts prey of variable species and size depending on local availabilities [33]. *N*. *clavipes* sexual behavior is polygamous with male-male competition and occasional sexual cannibalism [34].

### Study site

The present study was carried out in October 2014, in the locality of El Limón de Cuauchichinola, in Sierra de Huautla Biosphere Reserve, Morelos, Mexico (18°20’10” N, 98° 51’ 20” W). The study site is a tropical dry forest that comprises a mosaic of primary and secondary forest surrounded by agricultural land and towns. Despite the fact that half of the reserve is considered to be intact or in a good conservation status, the secondary forest in the reserve is used for wood extraction and extensive cattle and other livestock ranching, allowing animals to freely feed from the forest in ca. 20% of the Reserve area [35]. To maintain the coexistence of economic activities and biodiversity in the Reserve, a restoration experiment was set in January 2006; therefore, the age of the experimental plots at the time of the present study was eight years and 10 months.

The experimental design consists of eight 50 x 50 m restoration plots that were excluded from livestock with electric fences (minimal intervention). In addition to being excluded from disturbance, four plots excluded from livestock were planted with 560 plants each, belonging to 20 native tree species (maximal intervention [36]). Experimental plots are separated by 80–1600 m from each other, and plots from the same treatment are separated by at least 200 m. When compared to disturbed areas, both the minimal and maximal intervention treatments show signs of recovery in biomass and richness of herbs [37], dispersal processes [38], advanced regeneration [39], and lepidopteran diversity [40].As superior and inferior controls respectively, the restoration experiment uses three plots of conserved forest (old forest, never cut) and three plots of disturbed forest where cattle still graze. In summary, the experiment consists in 14 plots of four different treatments: four plots of minimal intervention excluded from cattle (excluded), four excluded and planted (planted), three superior controls (conserved) and three inferior controls (disturbed).

### Study design

We collected a total of 146 adult male and 143 adult female *Nephila clavipes* spiders in different sites as follows: in control conserved plots, N = 42 males and 33 females; in excluded plots, N = 36 males and 33 females; in planted plots, N = 32 males and 40 females; in control disturbed plots, N = 36 males and 37 females. At least 6 males or females were collected in each plot, except for one plot in the maximal intervention treatment where we only found 2 males and 3 females. To reduce edge effects and invasion from contiguous sites, spiders were never collected less than five meters from the border of any plot. This distance is reasonable because most *Nephila* males and females that disperse move only one meter and can remain in the same place for weeks [41,42]. Collected individuals were immediately stored in ethanol 70% for subsequent measurement of body size and physiological condition.

For each spider, we removed all the legs and took three physiological measurements: total dry mass, lipid mass, and muscular mass. These measurements are good indicators of an individual’s nutritional status because they are related to individual fitness, and as such, they have been suggested as important parameters for conservation studies [26,30,43]. For measurements of lipid content, animals were dried at 45°C, weighed (± 0.1 mg) and submerged 48 h in chloroform for fat extraction. Samples were re-desiccated and re-weighed, and the difference in weight before and after chloroform extraction was considered lipid content (see similar procedures in [21,44]). For measurement of muscle mass, lipid-free dry samples were placed 48 h in a 0.8 M solution of potassium hydroxide for muscle extraction. Samples were re-desiccated and re-weighed and the difference in dry weight was considered muscle mass (see similar procedures in [45,46]. Body size was measured from digital pictures of the spiders taken with a millimetric scale, analyzed in Image J 1.42. For females, we used the length of the first left leg as an estimator of body size [47]. For males, that frequently lose legs, we used body length, measured from the tip of the cephalothorax to the tip of the abdomen [47].

### Statistical analyses

We tested the effect of restoration treatments and two controls (conserved, planted, excluded, and disturbed) on four body condition variables of *Nephila*: body size, total dry mass, lipid mass, and muscle mass in both males and females. Analyses were carried out separately for males and females. We used general linear mixed models, including restoration treatment as a fixed factor and plot identity nested in treatment as a random effect [48,49] for each dependent variable. To analyze body dry mass, lipid mass, and muscle mass, we included the additive effect of body size in the model. Including body size as a covariate in linear models generates better predictors of real body condition in spiders and other animals, thus this analysis strategy is recommended when estimating body condition [24,50]. When the restoration treatment factor was significant, differences between treatments were explored with a priori contrasts. Model assumptions of normality of the residuals and homogeneity of variances were inspected with normal Q-Q plots and plots of fitted values versus residuals of the mixed models respectively [48]. The presence of outliers was inspected using boxplots [48]. Data on female body dry mass, lipid mass, and muscle mass were square root transformed to achieve normality and homogeneity of variances. Analyses were carried out in R software version 3.1.1 [51].

## Results

Males and females showed different trends in their physiological response to restoration treatments, but in general, individuals in planted plots showed the highest condition values in both sexes. Male and female body sizes were not affected by restoration treatment (Table 1).

**Table 1:** Effect of restoration treatment and body size on body condition of *Nephila clavipes* spiders. Significant differences are shown in bold.

Factor/Body condition	Body size	Body dry mass	Lipid content	Muscle content
**Treatment**				
Males	F_3,10_ = 0.56, P = 0.651	F_3,10_ = 9.23, **P = 0.003**	F_3,10_ = 0.62, P = 0.617	F_3,10_ = 1.62, P = 0.245
Females	F_3,10_ = 1.48, P = 0.278	F_3,10_ = 14.61, **P<0.001**	F_3,10_ = 13.24, **P<0.001**	F_3,10_ = 12.52, **P<0.001**
**Body size**				
Males	-	F_1,131_ = 550.4, **P<0.001**	F_1,131_ = 0.70, P = 0.403	F_1,131_ = 30.9, **P<0.001**
Females	-	F_1,128_ = 39.18, **P<0.001**	F_1,128_ = 20.73, **P<0.001**	F_1,128_ = 38.19, **P<0.001**

**Fig 1:**
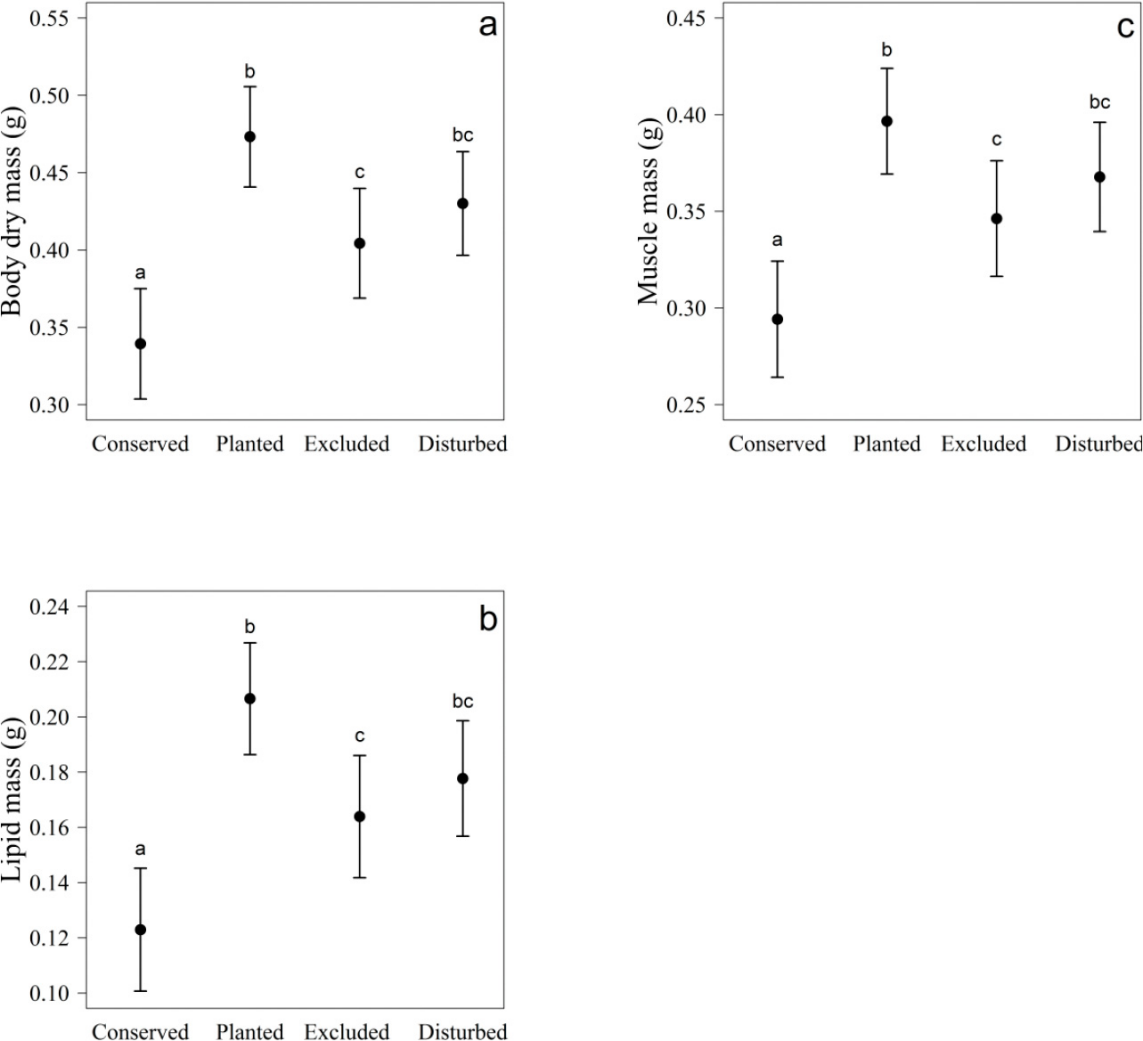
Effect of different habitats in body condition of female spiders. Body condition, measured as a) body dry mass, b) lipid mass, and c) muscle mass, of *Nephila clavipes* female spiders inhabiting plots under two levels of restoration (exclusions and plantings) and conserved and perturbed areas in the dry forest. Values in the y-axis are square root transformed. Different letters represent significant differences between treatments. Lines represent means ± 95% confidence intervals.

**Fig 2:**
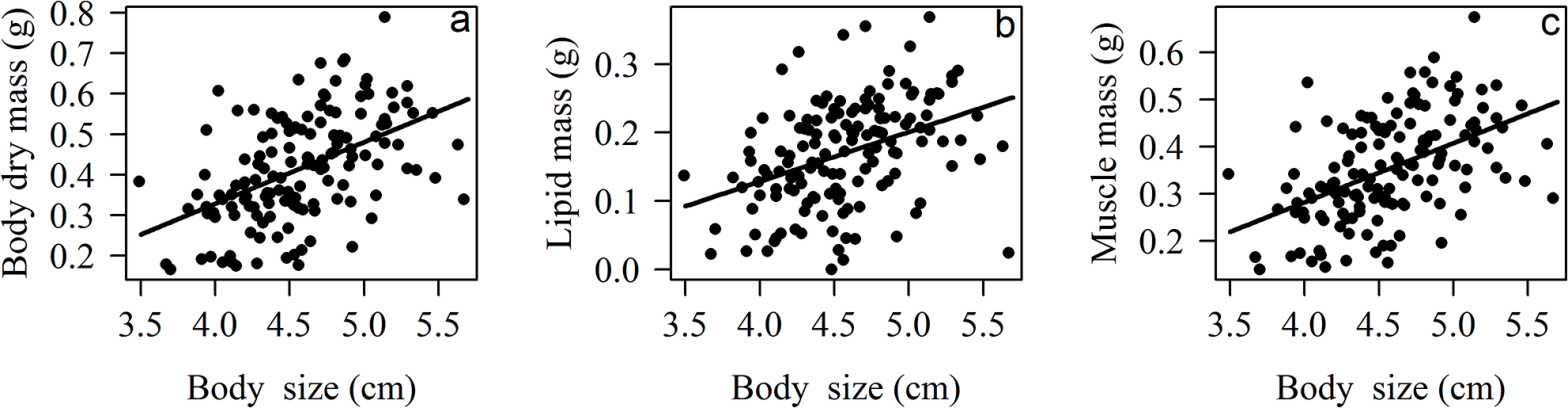
Relation between body size and body condition in *Nephila clavipes* female spiders. Condition was measured as a) body dry mass, b) lipid mass, and c) muscle mass. Data on the y axis are square root transformed.

In females, body dry mass, lipid and muscle mass were dependent on restoration treatment and body size (Table 1). In general, contrary to our predictions, female body condition (body, lipid, and muscle mass) was higher in disturbed than in conserved plots (Table 2, Fig 1A, 1B and 1C). As predicted, female body condition was higher in planted than in excluded plots (Table 2, Fig 1A, 1B and 1C). Females from excluded and disturbed plots did not differ in any of the physiological variables tested (Table 2). Body size was positively related with body, lipid and muscle mass (Fig 2A, 2B and 2C).

**Table 2:** A priori contrasts between different restoration treatments on indicators of body condition in *Nephila clavipes* spiders. When main effects are non significant (NS, see Table 1) comparisons are not shown. Significant differences are shown in bold.

	Conserved vs. Plantings	Conserved vs. Exclusions	Conserved vs. Disturbed	Plantings vs. Exclusions	Plantings vs. Disturbed	Exclusions vs. Disturbed
**Body size**						
Males	NS	NS	NS	NS	NS	NS
Females	NS	NS	NS	NS	NS	NS
**Body dry mass**						
Males	t = 2.72, **P = 0.022**	t = 0.85, P = 0.413	t = 0.07, P = 0.947	t = 1.83, P = 0.098	t = 2.67, **P = 0.023**	t = 0.89, P = 0.396
Females	t = 5.36, **P˂0.001**	t = 2.51, **P = 0.031**	t = 3.59, **P = 0.005**	t = 2.77, **P = 0.020**	t = 1.80, P = 0.102	t = 1.02, P = 0.330
**Lipid content**						
Males	NS	NS	NS	NS	NS	NS
Females	t = 5.36, **P˂0.001**	t = 2.54, **P = 0.029**	t = 3.48, **P = 0.006**	t = 2.76, **P = 0.020**	t = 1.93, P = 0.082	t = 0.88, P = 0.399
**Muscle content**						
Males	NS	NS	NS	NS	NS	NS
Females	t = 4.86, **P˂0.001**	t = 2.39, **P = 0.038**	t = 3.46, **P = 0.006**	t = 2.41, **P = 0.037**	t = 1.43, P = 0.183	t = 1.02, P = 0.333

**Fig 3:**
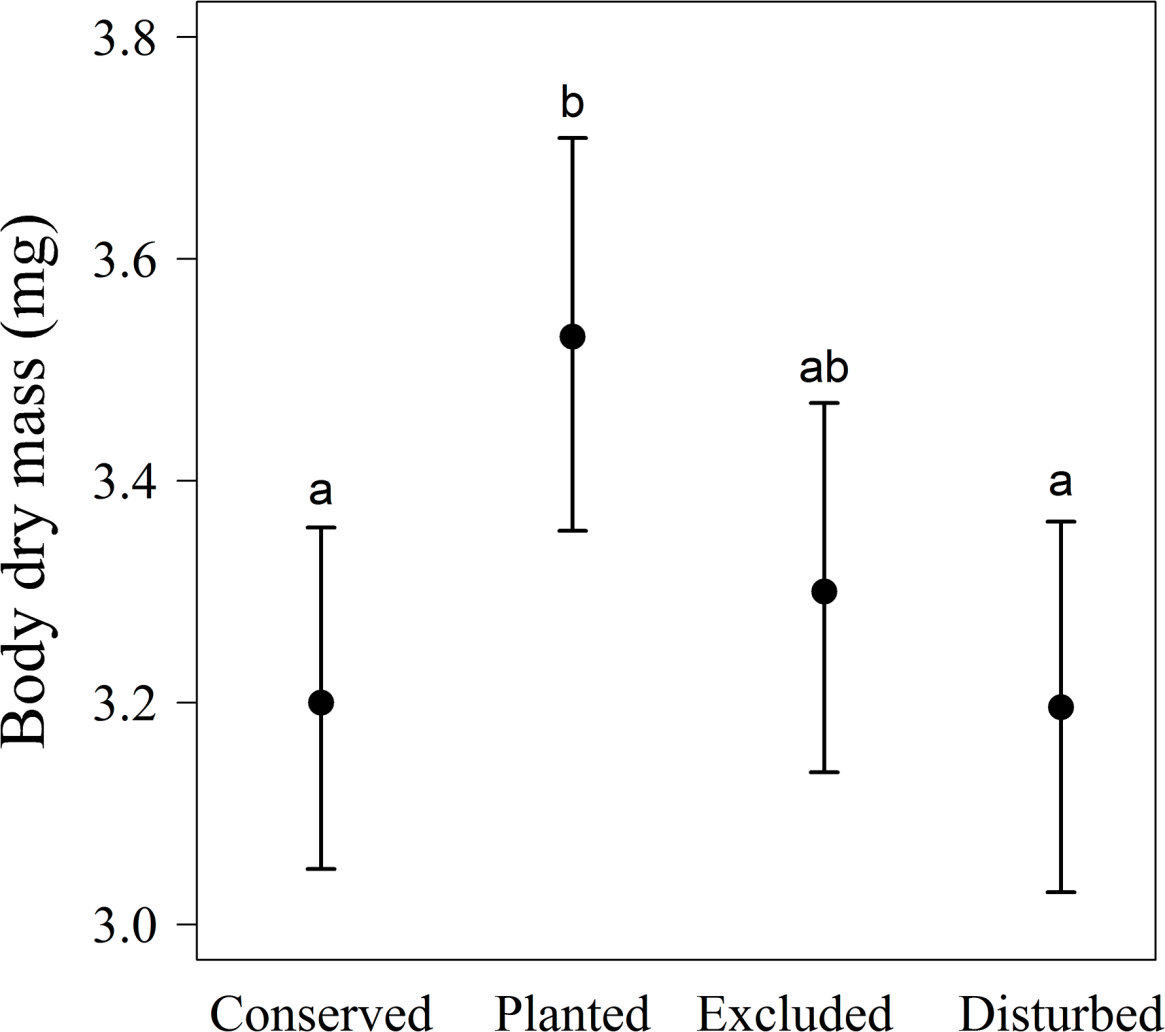
Effect of different habitats on body dry mass of *Nephila clavipes* male spiders inhabiting plots under two levels of restoration (exclusions and plantings) and conserved and perturbed areas in the dry forest. Different letters represent significant differences between treatments. Lines represent means ± 95% confidence intervals.

**Fig 4:**
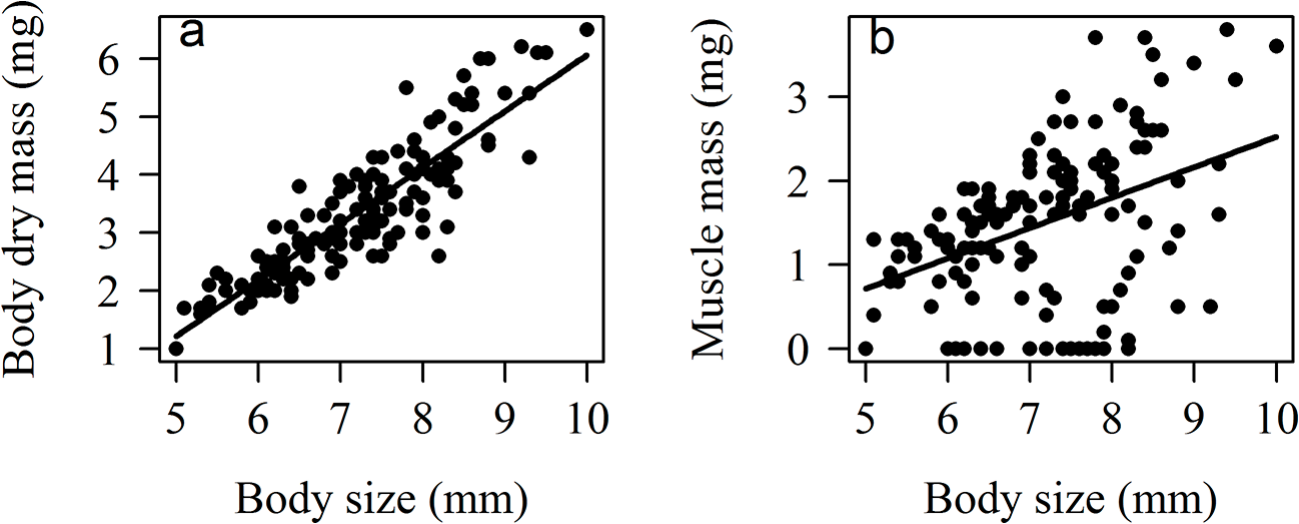
Relation between body size and body condition in *Nephila clavipes* male spiders. Body condition was measured as a) body dry mass, and b) muscle mass.

The trends observed in males did not follow our predictions. Body dry mass was affected by restoration treatment: males in plantings had heavier bodies than males in conserved or disturbed plots, but similar weights to those in excluded plots (Table 1; Fig 3). Contrary to our predictions, body dry mass did not differ between males from conserved and disturbed plots. Lipid mass and muscle mass did not differ across restoration treatments (Table 1). Body size was positively related with body dry mass and muscle mass, but not with lipid mass (Table 1; Fig 4A and 4B).

## Discussion

Success of ecological restoration programs has mainly been evaluated by the recovery of populations and communities, despite the attention called to the importance of evaluating individual physiology as a measure of restoration success [9]. In contrast with studies at population or community levels, physiology can reflect the quality of ecosystems in a shorter time scale, and represents the mechanistic basis of changes in populations and communities, which are fundamental in conservation programs [9,10]. In the present study, we evaluated for the first time how different restoration methods affect body condition of a terrestrial animal, the native spider *Nephila clavipes*. We studied body condition with respect to nutritional ecology, by measuring male and female body size and three key indicators of physiological condition. We predicted (1) lower general body condition in disturbed sites compared to restored or conserved sites, and (2) a better body condition when restoration included maximal intervention (plantings) than in sites with minimal intervention. Our first prediction was not supported in males or females, whereas our second prediction was supported only in females.

Individual body condition is largely dependent on nutritional status and thus on resource availability, which is typically low for spiders in nature [26]. Given their different life histories, male and female spiders have different nutritional requirements and are expected to perform differently on different diets [26]. The measurements of physiological condition used in the present study [body mass, lipid content and muscle mass] are dependent on resource availability and are good predictors of individual fitness. On one hand, lipids are a fundamental energy source for spiders [26]. Energy is obtained from food [28] and is mainly spent on functions including foraging, reproduction, metabolism, endocrine control, and immune response [52–54]. Therefore, lipids can mediate a number of life history trade-offs related with survival and reproduction [28,55]. On the other hand, muscles, which are mainly built of proteins [56], can be important for mating, foraging, and predation avoidance, so muscle mass can also be related directly to individual fitness [57,58]. Our contrasting results in males and females suggest that both have different nutritional requirements, and that lipid and protein availability are variable across the different environments evaluated.

Prey abundance, diversity, and nutritional quality can be the main drivers of physiological changes detected across our study sites in both sexes. We do not known the main diet resources for *N*. *clavipes* spiders in our study sites, however, prey availability could have large effects on spider performance [59,60], especially if, as other spiders, *N*. *clavipes* forage selectively for different nutrients to counteract specific nutrient imbalances [20,21]. Changes in the abundance and identity of prey species may differ between our studied sites, causing the observed effects on spider physiology, and potentially affecting populations, communities, or ecosystems [23]. In our experimental plots, Lepidopera abundance in plantings was similar to exclusions and disturbed sites, whereas richness was 20 times higher in restored compared to disturbed sites [40]. Other potential spider prey may follow similar community compositional and abundance changes across studied sites. Given that prey diversity plays a fundamental role in spider physiology, as different prey provide nutrients of different quality [60,61], our contrasting results in male and female spider physiology may reflect that the two sexes responded differently to prey diversity. Future research in our study sites should evaluate whether prey availability and quality vary for males and females across plots.

In females, our results supported our second prediction, but not the first: female spiders in planted and disturbed sites showed higher fat content as well as muscle mass and body mass compared to those inhabiting excluded and conserved sites. These results contrast with our findings in males, where individuals in conserved sites showed similar body condition to those inhabiting disturbed and excluded plots. However, body dry mass in males and females was higher in planted than in control conserved sites for both sexes, and higher than in disturbed plots for males, suggesting that plantings are providing more resources.

Male and female energetic requirements can be very different, as they allocate their energetic resources to different functions [26]. Probably, the main difference between male and female spiders in this respect is that females invest a large amount of lipids on egg production, whereas males invest more in mate searching or in competition for mates [24,62]. However, other ecological factors, such as competition for prey resources or parasitism, can differ between sexes. *N*. *clavipes* males and females compete for prey of different sizes [32], and parasites can have sex-differential development and virulence in their hosts [63], altering the host’s foraging behavior and consequently its body condition [46]. Future studies should evaluate the different dietary requirements for male and female spiders, as well as the presence of potential competitors or parasite pressures in the different studied environments in order to test whether these factors are impacting spider physiology and, to a larger extent, spider populations.

In contrast to our findings on physiological status, neither male nor female spiders differed in body size between the different experimental sites analyzed. Unlike physiological traits, that reflect immediate body condition [9], adult body size in arthropods is fixed and, despite having an important genetic component, can be largely dependent on environmental conditions experienced during early development [64,65]. Spiders have the ability to catch up on growth and development after surviving periods of nutritional imbalance, presumably to synchronize reproduction to a specific season [66]. Such compensatory growth can be achieved by increasing feeding rates when conditions improve, or by taking resources at the expense of other functions [i. e. physiological], which become depleted and can affect individuals later in life [66]. Hence, physiological changes found for spiders in our study sites might have resulted from a pressure experienced early in life to reach normal adult sizes. During juvenile stages, male and female *N*. *clavipes* spiders are similar in body size and both use orb-webs for hunting, thus they are likely feeding on very similar prey [32]. On the other hand, mature spiders are highly size-dimorphic, and males and females may not consume the same prey items, as males stop making their own webs and start looking for females [32]. At this stage, males either do not feed, or more likely ingest prey rejected by females or silk from female webs, which will enhance their longevity [67,68]. If adult males do not feed anymore, their physiological status at the time of maturation will depend on the conditions during development; if they do feed, their physiological status will depend on current conditions. Future studies evaluating male and female physiological status during development could show if our findings resulted from pressures during youth or from conditions during adulthood.

In the present study we measured a set of condition-dependent physiological traits in response to different environmental conditions. Given that physiology is a main driver of evolution [69], it is likely that evolutionary processes, such as sexual selection, can be changing in disturbed or restored sites. Sexual selection is highly dependent on sexual traits, such as morphologies and behaviors that are favored during mating, and are considered honest signals of individual quality [70]. Future studies should evaluate whether sexual selection processes, such as male-male competition in *N*. *clavipes* spiders [34], are different in disturbed and restored sites as a result of different environmental conditions, leading to changes in the evolutionary trajectories of populations in different habitats [28].

In *N*. *clavipes* spiders, alternative methods of ecological restoration had different impacts on male and female body condition. Our results highlight the importance of incorporating physiological studies in conservation biology, which could include evaluations of other key species such as pollinators, herbivores, pests, or decomposers, that strongly impact restoration efforts [7,71,72]. By using tools from nutritional ecology, physiology can help to clarify the proximal mechanisms that confer stability and function to restored ecosystems.
